# Completing sparse and disconnected protein-protein network by deep learning

**DOI:** 10.1186/s12859-018-2112-7

**Published:** 2018-03-22

**Authors:** Lei Huang, Li Liao, Cathy H. Wu

**Affiliations:** 1 Department of Computer and Information Sciences, University of Delaware, 18 Amstel Avenue, Newark, 19716 Delaware USA; 2 Center for Bioinformatics and Computational Biology, University of Delaware, 15 Innovation Way, Newark, 19711 Delaware USA

**Keywords:** Disconnected protein interaction network, Neural network, Interaction prediction, Network evolution, Regularized Laplacian

## Abstract

**Background:**

Protein-protein interaction (PPI) prediction remains a central task in systems biology to achieve a better and holistic understanding of cellular and intracellular processes. Recently, an increasing number of computational methods have shifted from pair-wise prediction to network level prediction. Many of the existing network level methods predict PPIs under the assumption that the training network should be connected. However, this assumption greatly affects the prediction power and limits the application area because the current golden standard PPI networks are usually very sparse and disconnected. Therefore, how to effectively predict PPIs based on a training network that is sparse and disconnected remains a challenge.

**Results:**

In this work, we developed a novel PPI prediction method based on deep learning neural network and regularized Laplacian kernel. We use a neural network with an autoencoder-like architecture to implicitly simulate the evolutionary processes of a PPI network. Neurons of the output layer correspond to proteins and are labeled with values (1 for interaction and 0 for otherwise) from the adjacency matrix of a sparse disconnected training PPI network. Unlike autoencoder, neurons at the input layer are given all zero input, reflecting an assumption of no a priori knowledge about PPIs, and hidden layers of smaller sizes mimic ancient interactome at different times during evolution. After the training step, an evolved PPI network whose rows are outputs of the neural network can be obtained. We then predict PPIs by applying the regularized Laplacian kernel to the transition matrix that is built upon the evolved PPI network. The results from cross-validation experiments show that the PPI prediction accuracies for yeast data and human data measured as AUC are increased by up to 8.4 and 14.9% respectively, as compared to the baseline. Moreover, the evolved PPI network can also help us leverage complementary information from the disconnected training network and multiple heterogeneous data sources. Tested by the yeast data with six heterogeneous feature kernels, the results show our method can further improve the prediction performance by up to 2%, which is very close to an upper bound that is obtained by an Approximate Bayesian Computation based sampling method.

**Conclusions:**

The proposed evolution deep neural network, coupled with regularized Laplacian kernel, is an effective tool in completing sparse and disconnected PPI networks and in facilitating integration of heterogeneous data sources.

## Background

Studying protein-protein interaction (PPI) can help us better understand intracellular signaling pathways, model protein complex structures and elucidate various biochemical processes. To aid discovering more denovo PPIs, many computational methods have been developed and can generally be categorized into one of the following three types: (a) pair-wise biological similarity based computational approaches by sequence homology, gene co-expression, phylogenetic profiles, three-dimensional structural information, etc.; [1–7]; (b) pair-wise topological features based methods [8–11]; and (c) whole network structure based methods [1, 12–20].

For the pair-wise biological similarity based methods, without resort to determining whether two given proteins will interact from first principles in physics and chemistry, the predictive power of those methods is greatly affected by the features being used, which may be noisy or inconsistent. To circumvent limitations of pair-wise biological similarity, network structure based methods are playing an increasing role in PPI prediction since these methods can not only get the whole network structure involved and topological similarities implicitly included, but also utilize pair-wise biological similarities as weights for the edges in the networks.

Along this line, variants of random walk [12–15] have been developed. Given a PPI network with *N* proteins, the computational cost of these methods increases by *N* times for all-against-all PPI prediction. In Fouss et al. [16], many kernel methods for link prediction have been systematically studied, which can measure the similarities for all node pairs and make prediction at once. Compared to the random walk, kernel methods are usually more efficient. However, neither random walk methods nor kernel methods perform very well in predicting interaction between faraway node pairs in networks [16]. Instead of utilizing network structure explicitly, many latent features based on rank reduction and spectral analysis have also been used to do prediction, such as geometric de-noise methods [1, 17], multi-way spectral clustering [18], matrix factorization based methods [19, 20]. Note that the objective functions in these methods should be carefully designed to ensure fast convergence and avoid being stuck in local optima. What is advantagous for these methods is that biological features and network topological features can complement each other to improve the prediction performance, such as by weighting network edges with pair-wise biological similarity scores [19, 20]. However, one limitation for these methods is that, only the pair-wise features for the existing edges in the PPI network are utilized, whereas from a PPI prediction perspective what is particularly useful is to incorporate pair-wise features for node pairs that are not currently linked by a direct edge but may become linked. Recently, Huang et al. proposed a sampling method [21] and a linear programming method [22] to find optimal weights for multiple heterogeneous data, thereby building weighted kernel fusion for all node pairs. These methods applied regularized Laplacian kernel (RL) to the weighted kernel fusion to infer missing or new edges in the PPI network. These methods improved PPI prediction performance, especially for detecting interactions between nodes that are far apart in the training network, by using only small training networks.

However, almost all the methods discussed above need the training network to be a single connected component to measure node-pair similarities, despite of the fact that existing PPI networks are usually disconnected. Consequently, these traditional methods only keep the maximum connected component of the original PPI network as golden standard data, which is then divided as a connected training network and testing edges. That is to say, these methods cannot effectively predict interactions for proteins that are not located in the maximum connected component. Therefore, it is of great interest and utility if we can infer PPI network from a small amount of interaction edges that do not need to form a connected network.

From our previous study of network evolutionary analysis [23], we here designed a neural network based evolution model to implicitly simulate the evolution processes of PPI networks. Instead of simulating the evolution of the whole network structure with the growth of nodes and edges as models discussed in Huang et al. [23], we only focus on the edge evolution and assume all nodes are already existing. We initialize the ancient PPI network as an all-zero adjacent matrix, and use the disconnected training network with interaction edges as labels. Each row of the all-zero adjacent matrix and the training matrix will be used as the input and label for the neural network respectively. We then train the model to simulate the evolution process of interactions. After the training step, we use outputs of the last layer of the neural network to represent rows of the evolved contact matrix. Finally, we further apply the regularized Laplacian kernel to a transition matrix that is built upon the evolved contact matrix to infer new PPIs. The results show our method can efficiently utilize the extremely sparse and disconnected training network, and improve the prediction performances by up to 8.4% for yeast and 14.9% for human PPI data.



## Methods

### Problem definition

Formally, a PPI network can be represented as a graph *G*=(*V*,*E*) where *V* is the set of nodes (proteins) and *E* is the set of edges (interactions). *G* is defined by the adjacency matrix *A* with |*V*|×|*V*| dimension:

1 $$ {A(i,j)} = \left\{ \begin{array}{c} 1, if {(i,j)}\in{E} \\ 0, if {(i,j)}\notin{E} \\ \end{array} \right.  $$

where *i* and *j* are two nodes in the nodes set *V*, and (*i*,*j*) represents an edge between *i* and *j*, (*i*,*j*)∈*E*. We divide the golden standard network into two parts: the training network *G*_*tn*_=(*V*,*E*_*tn*_), and testing set *G*_*tt*_=(*V*_*tt*_,*E*_*tt*_), such that *E*=*E*_*tn*_∪*E*_*tt*_, and any edge in G can only belong to one of these two parts. The detailed process of dividing the golden standard network is shown by Algorithm 1. We set the *α* (the preset ratio of *G*_*tn*_(,*E*) to *G*(,*E*)) less than a small value to make the *G*_*tn*_ extremely sparse and with a large number of disconnected components.

**Fig. 1:**
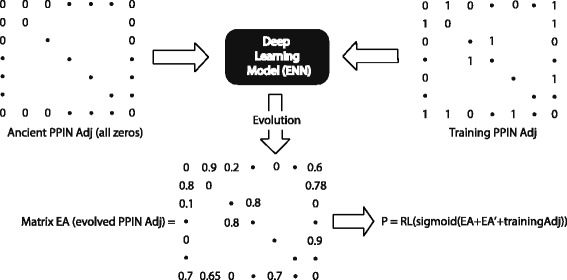
The flow chart of *ENN*−*RL* method

**Fig. 2:**
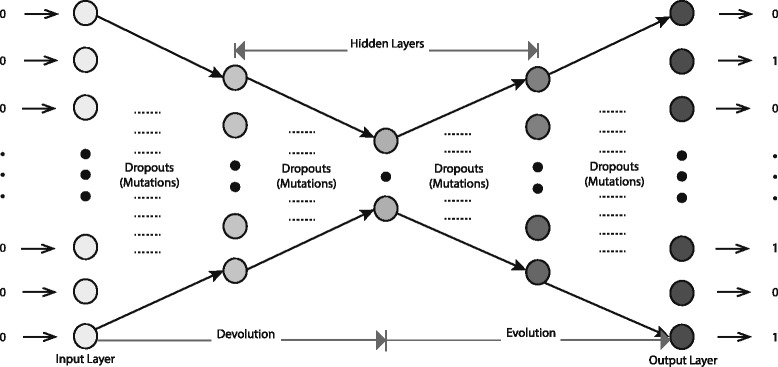
The evolution neural network *ENN*

Figure 1 shows the flow chart of our method, which is named as evolution neural network based regularized Laplcian kernel (ENN-RL) to reflect the fact that it contains two steps. The first step, *ENN*, uses the sparse disconnected training network of PPIs to train a deep neural network in order to obtain an “evolved” and more complete network, and this “evolved” network is then used as a transition matrix for the regularized Laplacian kernel in the second step to predict PPIs for node pairs that are not directed connected. Inspired by the structure of autoencoder [24], the architecture of the neural network is designed to “evolve” a partial PPI network, guided by its current connection, via a smaller proteome (i.e., a smaller hidden layer) at an “ancient” time, asssuming zero a priori knowledge of existing PPIs at the input. Specifically, as shown in Fig. 2, with respect to the smaller hidden layer in the middle, the input layer looks like a symmetric mirror image of the output layer. But all nodes in the input layer have zero value input, reflecting the assumption of zero a priori knowledge about interactions between proteins that these nodes represent, i.e., the input *m*×*m* adjacency matrix in Fig. 1 contains all zeros, where *m*=|*V*|. And the output layer nodes have labels from the training PPI adjacency matrix. Then, deep learning is adopted to drive and guide the neural network from a blank input to first “devolve” into a smaller hidden layer (representing an interactome at ancient time) and then “evolve” into the output layer, which has the training PPI network *G*_*tn*_ as the target/label. The rationale is that, if PPI interactions in the training network can be explained (or reproduced) via a smaller ancient interactome, the such trained neural network should be also capable of generalizing and predicting unobserved de novo PPIs. To avoid exactly producing the training PPI networks, a blank adjacency matrix is used as the input.

After the training process is completed, we build the evolved PPI network/matrix *EA* with the outputs of neural network’s last layer. Based on *EA*, we build a transition matrix using Eq. (2), where *EA*+*EA*^′^ makes the transition matrix symmetric and positive semi-definite. Finally, we apply the regularized Laplacian (RL) kernel defined by Eq. (3) to the transition matrix *T* to get the inference matrix *P*, in which *P*_*i*,*j*_ indicates the probability of an interaction for protein *i* and *j*. For Eq. (3), *L*=*D*−*T* is the Laplacian matrix made of the transition matrix *T* and the degree matrix *D*, and 0<*α*<*ρ*(*L*)^−1^ and *ρ*(*L*) is the spectral radius of *L*.

2 $$ \textit{T} = sigmoid(EA'+EA+trainingAdj)  $$

3 $$ \textit{RL} = \sum\limits_{k=0}^{\infty} \alpha^{k}{(-L)}^{k} = {(I+\alpha*L)}^{-1}  $$

Algorithm 2 describes the detailed training and prediction processes.



### Evolution neural network

The structure of the evolution neural network is shown in the Fig. 2, which contains five layers including the input layer, three hidden layers and the output layer. Sigmoid is adopted as the activation function for each neuron, and layers are connected with dropouts. Dropouts can not only help us prevent over-fitting, but also indicate the mutation events during the evolution processes, such as which nodes (representing proteins) at a layer (corresponding a time during evolution) may be evolved from some nodes from the previous layer, as indicated by edges and corresponding weights connecting those nodes.

For specific configuration of the neural network in our experiments, the number of neurons in the input and out-put layer depends on the network size *m*=|*V*| of specific PPI data. Each protein is represented by the corresponding row of the adjacency matrix *trainingAdj* of *G*_*tn*_ that contains the interaction information for that protein with other proteins in the proteome. We train the evolution neural network by each row of the blank adjacency matrix as the input and the corresponding row of *trainingAdj* as the label. A typical autoencoder structure is chosen for the three hidden layers, where encoder and decoder correspond to the biological devolution and evolution processes respectively; and cross entropy is used as the loss function. Note that, the correspondence of encoder/decoder to biological devolution/evolution is at this stage more of an analogy in helping with the design of the neural network structure than a real evolution mode for PPI networks. It is also worth noting that different with the traditional autoencoder, we did not include the layerwise isomorphism pretraining to initial the weights for our neural network since the inputs are all zero vectors. The neural network is implemented by the TensorFlow library [25], deployed on Biomix cluster at Delaware Biotechnology Institute.

### Data

We use yeast and human PPI networks downloaded from DIP (Release 20140117) [26] and HPRD (Release 9) [27] to train and test our method. After removing the self-interactions, the detailed information of these two datasets are shown in the Table 1.

**Table 1:** PPI network information

Species	Proteins	Interactions
Yeast	5093	22,423
Human	9617	37,039

## Results

### Experiments on yeast PPI data

To show how well our model can predict PPIs from the extremely sparse training network with disconnected components, we set *α*, the ratio of interactions in *G*_*tn*_ to the total edges in *G*, to be less than 0.25. As shown in Table 2, the *G*_*tn*_ has only 4061 interactions, and contains 2812 disconnected components, where the minimum, average and maximum size of components are 1, 1.81 and 2152 respectively. Based on the *G*_*tn*_, we train our model and predict the large testing set *G*_*tt*_ that has 18,362 interactions according to the Algorithm 2.

**Table 2:** Division of yeast golden standard PPI interactions

*α*	*G*_*tn*_	*G*_*tn*_(*#C*)	*G*_*tn*_(*minC*,*avgC*,*maxC*)	*G*_*tt*_
0.25	4061	2812	(1, 1.81, 2,152,)	18,362
0.125	1456	3915	(1, 1.30, 1,006)	20,967

*G*_*tn*_(*#C*): the number of components in *G*_*tn*_

*G*_*tn*_(*minC*,*avgC*,*maxC*): the minimum, average and maximum size of components in *G*_*tn*_

**Fig. 3:**
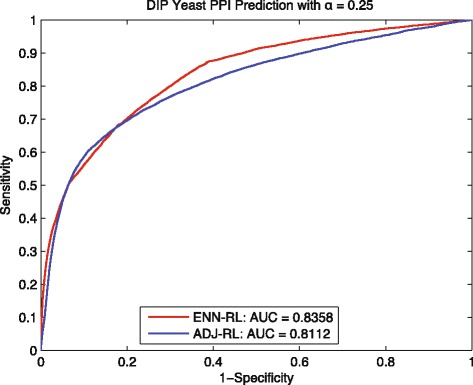
Yeast: ROC curves of predicting *G*_*tt*_∼18362 with *α*=0.25

**Fig. 4:**
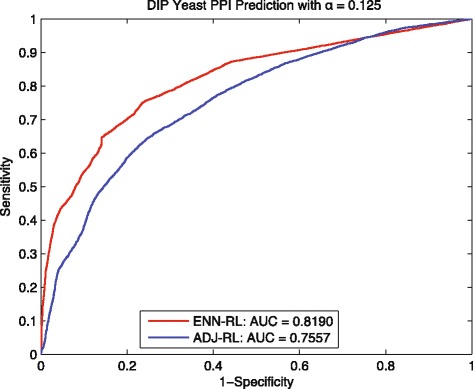
Yeast: ROC curves of predicting *G*_*tt*_∼20967 with *α*=0.125

We then compared our ENN-RL method to the control method ADJ-RL which applies regularized Laplacian kernel directly to the training network *G*_*tn*_. As shown in Fig. 3, the AUC increase from 0.8112 for the control method to 0.8358 for ENN-RL. Moreover, we make the prediction task more challenging by setting the *α* to be less than 0.125, which makes the *G*_*tn*_ sparser with only 1456 interactions, but 3915 disconnected components; and the maximum component in *G*_*tn*_ only has 1006 interactions. The results in Fig. 4 shows the gap between ENN-RL ROC curve and ADJ-RL ROC curve is obviously increased; and our ENN-RL gained 8.39% improvement in AUC. If comparing Figs. 3 and 4, it is easy to see that the AUC of ADJ-RL decreases by 0.055 from 0.8112 in Fig. 3 to 0.7557 in Fig. 4. However, our ENN method performs stably with only 0.016 decrease in AUC. This suggests that traditional random walk methods usually need the training network to be connected; and the prediction performance largely depends on the size and density of the maximum connected component. However, when the training network becomes sparse and disconnected, the traditional random walk based methods will lose the predictive power likely because they cannot predict interactions among those disconnected components. We repeated the whole experiments up to ten times, Table 3 shows the average performance with the standard deviation. All these results show our method performs stably and effectively in overcoming the limitation of traditional random walk based methods; and the improvements are statistically significant.

**Table 3:** AUC summary of repetitions for yeast PPI data

Methods	Avg ± Std (*α*=0.25)	Avg ± Std (*α*=0.125)
ENN-RL	0.8339 ± 0.0016	0.8195 ± 0.0023
ADJ-RL	0.8104 ± 0.0039	0.7403 ± 0.0083

**Fig. 5:**
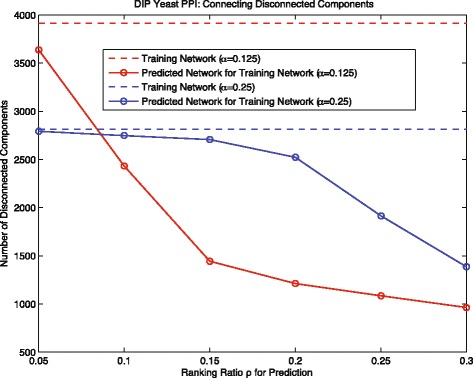
Yeast: connecting disconnected components

Moreover, we further analyzed how our ENN-RL method can effectively predict interactions to connect disconnected components, and how its intra-component and cross-component predicting behaviors adaptively change with different training networks. As defined in the “Methods” section, the value *P*_*i*,*j*_ in the inference matrix *P* indicates the probability of an interaction for protein *i* and *j*. We ranked all the protein pairs by their value in the inference matrix *P*; ideally we can choose a optimal threshold on the value of *P*_*i*,*j*_ to make prediction. Since it is difficult to find the optimal threshold without prior knowledge, we used a ratio *ρ* instead. Specifically, for the ranked protein pairs, the top *ρ*∗100 percent can be considered as predicted positives *G*_*pp*_, and the predicted true positive *G*_*ptp*_ is the intersection set between *G*_*pp*_ and *G*_*tt*_. We then added the interactions in *G*_*ptp*_ to the training network *G*_*tn*_ to see how many disconnected components can become reconnected. The results are shown in the Fig. 5, the dashed lines indicate the number of disconnected components in the training networks *G*_*tn*_; the solid lines with markers indicate that how the number of disconnected components would change based on prediction with different *ρ* (The red color is for the case *α*=0.125, the blue color is for *α*=0.25). As it shows, our methods can effectively predict interactions to reconnect those disconnected components in the training networks for both cases; especially, for the training network of *α*=0.125. The comparison of the results of those two cases shows that the red solid line decreases significantly faster than the blue solid line. It demonstrates that, for the training network *α*=0.25 that has fewer but larger size disconnected components, the prediction of our method recovers more intra-component interactions; whereas for the training network *α*=0.125 that has more and smaller size disconnected components, the prediction of our method can more effectively recover cross-component interactions, which are more difficult for the traditional random walk methods to detect. Therefore, to a large extent, this explains why the performance difference between our method and the traditional random walk method ADJ-RL is relatively small in Fig. 3 but more pronounced in Fig. 4, because in the latter case our method has clear advantage in detecting cross-component interaction.

### Experiments on human PPI data

We further tested our method by the human PPI data downloaded from HPRD (Release 9) [27], which is much larger and sparser than the yeast PPI network. Similarly, we carried out two comparisons by setting the *α* to be less than 0.25 and 0.125 respectively to divide *G* in to training network *G*_*tn*_ and testing set *G*_*tt*_. The detailed information about the division can be found in the Table 4.

**Table 4:** Division of human golden standard PPI interactions

*α*	*G*_*tn*_	*G*_*tn*_(*#C*)	*G*_*tn*_(*minC*,*avgC*,*maxC*)	*G*_*tt*_
0.25	6567	5370	(1, 1.79, 3,970)	30,472
0.125	2260	7667	(1, 1.25, 1,566)	34,779

*G*_*tn*_(*#C*): the number of components in *G*_*tn*_

*G*_*tn*_(*minC*,*avgC*,*maxC*): the minimum, average and maximum size of components in *G*_*tn*_

**Fig. 6:**
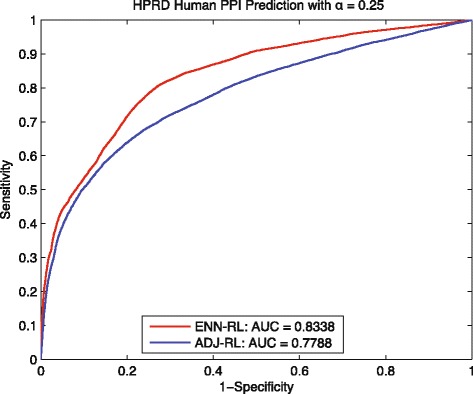
Human: ROC curves of predicting *G*_*tt*_∼30742 with *α*=0.25

**Fig. 7:**
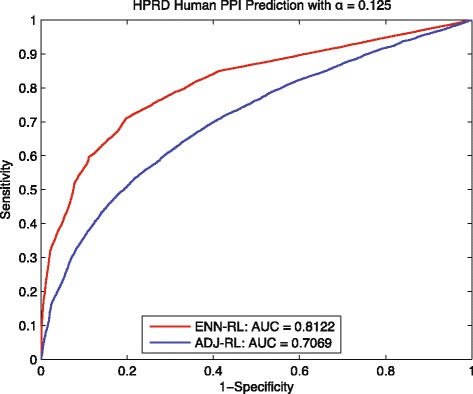
Human: ROC curves of predicting *G*_*tt*_∼34779 with *α*=0.125

The prediction performances in Figs. 6 and 7 show our ENN-RL has obviously better ROC curves and higher AUC than that of ADJ-RL. Especially for the test with *α*=0.125, our ENN-RL method obtains up to 14.9*%* improvement for predicting 34,779 testing interactions based on a training set *G*_*tn*_ with only 2260 interactions but 7667 disconnected components. Similar tendency is also observed from Figs. 6 and 7. When *α* is decreased from 0.25 to 0.125, the AUC of ADJ-RL decreases by up to 0.072, while our ENN-RL only decreased by 0.021. We also did ten repetitions as shown in Table 5 to demonstrate the stable performance of the ENN-RL. All these results on human PPI data further indicate our ENN-RL model is a promising tool to predict edges for any sparse and disconnected training network.

**Table 5:** AUC summary of repetitions for human PPI data

Methods	Avg ± Std (*α*=0.25)	Avg ± Std (*α*=0.125)
ENN-RL	0.8320 ± 0.0012	0.8140 ± 0.0013
ADJ-RL	0.7795 ± 0.0047	0.6970 ± 0.0059

**Fig. 8:**
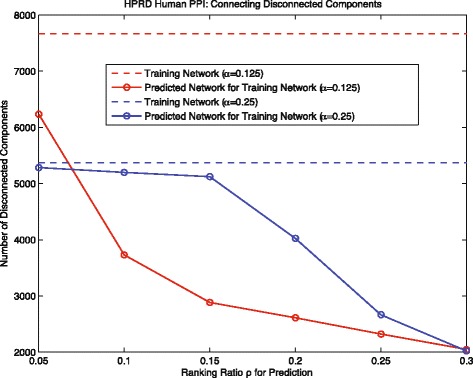
Human: connecting disconnected components

Moreover, similar to the experiments we did for the yeast data, we also analyzed the cross-component interaction prediction performance on HPRD human data. The result shown in the Fig. 8 is consistent with the result of yeast data. Our method can effectively predict interactions to connect disconnected components in both training networks (*α*=0.125 and *α*=0.25); and the red solid line decrease remarkably faster than the blue solid line. All these results further support the conclusion we made in the last section.

### Optimize weights for heterogeneous feature kernels

Most recently, Huang et al. [22, 28] developed a method to infer *de novo* PPIs by applying regularized Laplacian kernel to a kernel fusion that based on optimally weighted heterogeneous feature kernels. To find the optimal weights, they proposed weight optimization by linear programming (WOLP) method that based on random walk over a connected training networks. Firstly, they utilized Barker algorithm and the training network to construct a transition matrix which constrains how a random walk would traverse the training network. Then the optimal kernel fusion can be obtained by adjusting the weights to minimize the element-wise difference between the transition matrix and the weighted kernels. The minimization problem is solved by linear programming.

Given a large disconnected network, although Huang et al. [22] demonstrated that the weights learned from the maximum connected component can also be used to build kernel fusion for that large disconnected network, the weights will not be optimal when the maximum connected component is very small compared to the original disconnected network. As we all know that current available golden standard PPI networks are usually disconnected and remains far from complete. Therefore, it would be of great interest if we can obtain the transition matrix directly from these disconnected components, including but to limited to the maximum connected component, and use that transition matrix to help us find the optimal weights for heterogeneous feature kernels. To verify this idea, we use the transition matrix *T* obtained by Eq. (2) to find the optimal weights based on the linear programming Eq. (4) [22].

4 $$ W^{*}= \mathop{argmin}_{W}\left\| \left(W_{0}G_{tn} + \sum\limits_{i=1}^{n} W_{i}K_{i}\right) - T\right\|^{2}  $$

We tested this method by the yeast PPI network with same setting in Table 2; and six feature kernels are included: *G*_*tn*_: *G*_*tn*_ is training network with *α*=0.25 or 0.125 in Table 2. *K*_*Jaccard*_ [29]: This kernel measure the similarity of protein pairs *i*,*j* in term of $\frac {neigbors(i) \cap neighbors(j)}{neighbors(i) \cup neighbors(j)}$.

*K*_*SN*_: It measures the total number of neighbors of protein *i* and *j*, *K*_*SN*_=*neighbors*(*i*)+*neighbors*(*j*). *K*_*B*_ [30]: It is a sequence-based kernel matrix that is generated using the BLAST [31]. *K*_*E*_ [30]: This is a gene co-expression kernel matrix constructed entirely from microarray gene expression measurements. *K*_*Pfam*_ [30]: Similarity measure derived from Pfam HMMs [32]. All these kernels are normalized to the scale of [ 0,1] in order to avoid bias.

## Discussion

**Fig. 9:**
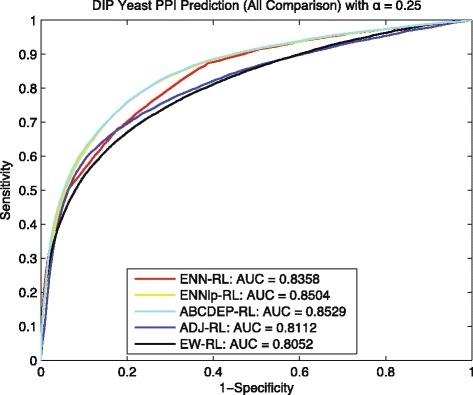
Yeast: ROC curves of predicting *G*_*tt*_∼18362 with *α*=0.25

**Fig. 10:**
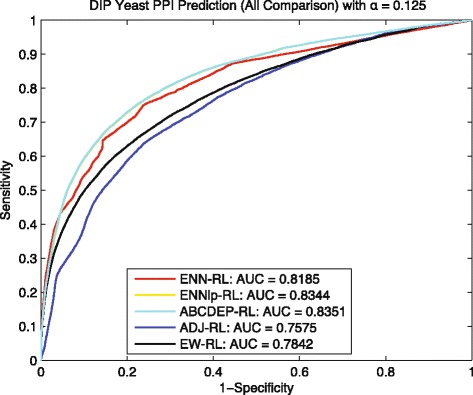
Yeast: ROC curves of predicting *G*_*tt*_∼20967 with *α*=0.125

To make a comprehensive analysis, we also included prediction results based on the kernel fusion built by the approximate bayesian computation and modified differential evolution sampling (ABCDEP) method [21], and the kernel fusion built by equally weighted feature kernels EK for comparison. Similar to the comparison in [22], the ABCDEP and EK based results can serve as the upper bound and lower bound of the prediction performance. Comparisons for two settings *α*=0.25 and *α*=0.125 are shown by the Figs. 9 and 10 respectively, where ENN-RL is our proposed method without integrating any heterogeneous feature kernels; ENNlp-RL is a kernel fusion method and is a combination of ENN-RL and the linear programming optimization method WOLP [22], which use the transition matrix *T* obtained by ENN-RL as the target transition matrix to find the optimal weights for heterogeneous feature kernels by linear programming; ABCDEP-RL is also a kernel fusion method and finding the optimal weights based on a sampling method [23]; ADJ-RL is the traditional random walk method; and EW-RL is a baseline kernel fusion method that weights all heterogeneous feature kernels equally. Note that, the transition matrix *T* obtained from ENN-RL is not a feature kernel and only serve as the target transition matrix for ENNlp-RL to optimize weights.

As shown in Fig. 9, ENNlp-RL benefits from a more complete and accurate target transition matrix provided by ENN-RL, outperforms other methods and gets very close to the upper bound 0.8529 achieved by ABCDEP-RL. Similarly, in Fig. 10, although the maximum component of *G*_*tn*_ is very sparse – with 1006 proteins and only 1456 training interactions, the ENNlp-RL still is enhanced from the ENN-RL and gets very close to the ABCDEP-RL. Therefore, all these results indicate that the transition matrix *T* learned by our *ENN* model can further improve the prediction performance for other downstream tools like WOLP in leveraging useful information from heterogeneous feature kernels.

## Conclusions

In this work we developed a novel method based on deep learning neural network and regularized Laplacian kernel to predict de novo interactions for sparse and disconnected PPI networks. We built the neural network with a typical auto-encoder structure to implicitly simulate the evolutionary processes of PPI networks. Based on the supervised learning using the rows of a sparse and disconnected training network as labels, we can obtain an evolved PPI network as the outputs of the deep neural network, which has an input layer identical to the output layer but with zero input value and a smaller hidden layer simulating an ancient interactome. Then we predicted PPIs by applying regularized Laplacian kernel to the transition matrix built upon that evolved PPI network. Tested on DIP yeast PPI network and HPRD human PPI network, the results show that our method exhibits competitive advantages over the traditional regularized Laplacian kernel that based on the training network only. The proposed method achieved significant improvement in PPI prediction, as measured by ROC score, over 8.39*%* higher than the baseline for yeast data, and 14.9*%* for human data. Moreover, the transition matrix learned from our evolution neural network can also help us to build optimized kernel fusion, which effectively overcome the limitation of traditional WOLP method that needs a relatively large and connected training network to obtain the optimal weights. Then we also tested it by the DIP yeast data with six feature kernels, the prediction result shows the AUC can be further improved and very close to the upper bound. Given the current golden standard PPI networks are usually disconnected and very sparse, we believe our model provides a promising tool that can effectively utilize disconnected networks to predict PPIs. In this paper, we designed the autoencoder deep learning structure analogous to the evolution process of PPI network, which, although should not be interpreted as a real evolution model of PPI networks, would nonetheless be worthwhile to explore further for the future work. Meanwhile, we also plan to investigate other deep learning models for solving PPI prediction problems.

## Abbreviations

ABCDEP: Approximate bayesian computation and modified differential evolution sampling
ADJ-RL: Adjacency matrix based regularized Laplacian kernel
AUC: Area under the curve
ENN: Evolution neural network
ENN-RL: Evolution neural network based regularized Laplacian kernel
PPI: Protein- protein interaction
RL: Regularized Laplacian kernel
ROC: Receiver operating characterisitic
WOLP: Weight optimization by linear programming
